# Influence of Gender and Reproductive Factors on Liver Fibrosis in Patients With Chronic Hepatitis B Infection

**DOI:** 10.14309/ctg.0000000000000085

**Published:** 2019-10-23

**Authors:** Ming Xiong, Junying Li, Shuling Yang, Fansen Zeng, Yali Ji, Jiang Liu, Qiaoping Wu, Qingjun He, Ronglong Jiang, Fuyuan Zhou, Weiqun Wen, Jinjun Chen, Jinlin Hou

**Affiliations:** 1 Hepatology Unit, Department of Infectious Diseases, Nanfang Hospital, Southern Medical University, Guangzhou, China;; 2 Department of Infectious Diseases, Guangzhou Women and Children's Medical Center, Guangzhou, China.

## Abstract

**INTRODUCTION:**

The role of reproductive factors in the development of chronic hepatitis B (CHB) remains unknown. We assessed the potential contributions of gender, menopausal status, and menarche age to liver fibrosis in CHB.

**METHODS:**

A cross-sectional prospective study included 716 women and 716 age-matched men with CHB who were not currently receiving antiviral therapy. Liver stiffness measurement using transient elastography was used to stage liver fibrosis as F0–F1 (<7.2 kPa), F ≥ 2 (7.2 kPa), F ≥ 3 (9.4 kPa), and F = 4 (12.2 kPa). Female patients were asked regarding their age at menarche and menopausal status using a questionnaire.

**RESULTS:**

Of the 716 women, 121 (16.9%) were postmenopausal, and 80 (11.2%) had advanced liver fibrosis. Multivariate logistic regression analysis showed that the postmenopausal status compared with the premenopausal status (odds ratio [OR] = 3.65–8.83; *P* < 0.05) and age at menarche of >14 years compared with <13 years (OR = 2.85–3.95; *P* < 0.05) were significantly associated with advanced fibrosis. Compared with premenopausal women, age-matched men had a higher OR for advanced fibrosis (*P* < 0.05). Compared with postmenopausal women, age-matched men did not show a significant difference in the degree of liver fibrosis (*P* > 0.05). Longitudinal data analysis showed that postmenopausal women (n = 31) were significantly less likely to undergo regression of liver fibrosis after antiviral treatment vs premenopausal women (n = 19) (26.3% vs 74.2%, respectively; *P* < 0.001).

**DISCUSSION:**

Menopause and late menarche aggravated liver fibrosis in untreated CHB, besides menopause delayed fibrosis regression under antiviral therapy. The protective effect of female gender against fibrosis was lost for postmenopausal women.

**TRANSLATIONAL IMPACT:**

It is important to consider menopausal status and age at menarche in establishing surveillance strategies among CHB females. Postmenopausal estrogen therapy may be considered for the prevention or treatment of liver fibrosis.

## INTRODUCTION

Menopause represents a state of increasing estrogen deficiency. There is evidence that menopause may increase the severity of liver fibrosis in the setting of hepatitis C virus (HCV) infection (1–3) and nonalcoholic fatty liver disease (NAFLD) (4). Increased duration of estrogen deficiency has been shown to be associated with an increased risk of liver fibrosis in NAFLD (5). Furthermore, hormone replacement therapy during menopause is associated with a reduced risk of liver fibrosis in patients with chronic HCV infection (1, 2). In a zebrafish model of experimental steatosis, ovarian senescence significantly increased the risk of severe liver fibrosis (6).

However, the relationship between menopausal status and liver fibrosis in chronic hepatitis B (CHB) remains to be investigated. Infection with hepatitis B virus (HBV) is an important global public health problem with significant morbidity and mortality (7). The progression of CHB depends on several host and environmental factors, including old age and male gender, which are recognized to be independent risk factors for the progression of liver disease (8, 9). Interestingly, the results from a previously published study demonstrated that the protective effect of female gender against HBV-associated cirrhosis was gradually lost after the age of 50 years (10), which is the average age of menopause in women in China (11).

Studies investigating the pathogenesis of HBV infection in animal models showed that the estrogen pathway could inhibit the viral transcription of HBV (12). A previous study also showed that an earlier onset of menarche was associated with earlier development of spontaneous hepatitis B e-antigen (HBeAg) seroconversion (13). These results support the influence of changes in female sex hormones on the pathogenesis of CHB.

Based on the findings from these previous studies, it may be proposed that menopause and late menarche may affect the progression of liver fibrosis in women with CHB. Transient elastography (TE) is a noninvasive method used to assess the degree of liver fibrosis. The diagnostic accuracy of TE has been validated in patients with CHB (14). TE can be used to quantify both the severity of liver fibrosis and its regression after antiviral treatment in terms of the liver stiffness measurement (LSM), which has been validated by biopsy-proven regression of fibrosis (15). The use of TE provides new possibilities for undertaking clinical research on liver fibrosis. Therefore, this study aimed to determine the influence of gender and reproductive factors on liver fibrosis in women with CHB.

## METHODS

### Study population and design

A prospective cross-sectional study was undertaken at the Department of Infectious Diseases, Nanfang Hospital, Guangzhou, China. Consecutive patients with CHB were recruited between June 2016 and March 2017.

Inclusion criteria were: (i) patients older than 18 years; (ii) serum hepatitis B surface antigen positive at least 6 months; (iii) not receiving antiviral therapy within the prior 1 year at the time of recruitment; and (iv) with valid TE.

Exclusion criteria were: (i) other causes of liver disease except for alcohol or NAFLD; (ii) history of liver transplant or hepatocellular carcinoma; (iii) hepatic decompensation; (iv) patients with alanine aminotransferase (ALT) >200 U/L; (v) malignant disease; and (vi) women without menopause status. The study was approved by the Institutional Review Boards of the Nanfang Hospital. We obtained written informed consent from each subject.

**Figure 1.**
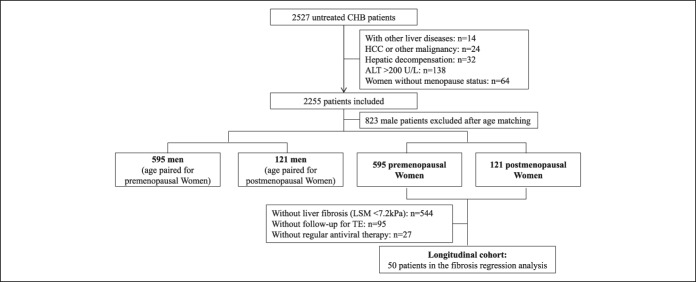
Flowchart of the study population. ALT, alanine aminotransferase; CHB, chronic hepatitis B; HCC, hepatocellular carcinoma; LSM, liver stiffness measurement; TE, transient elastography.

The study cohort was divided into 2 groups (Figure 1). Study participants who met the inclusion criteria included 716 women who were age-matched with 716 men in a 1:1 ratio for prevalence analysis. Of the women who were found to have liver fibrosis on repeat TE, 50 women who began regular antiviral therapy were included in the longitudinal study between June 2016 and October 2018.

### Outcome measures

Liver fibrosis was assessed by LSM using TE. The FibroScan 502 (EchoSens, Paris, France) was operated by 2 experienced ultrasonographers, and the results were expressed as kilopascals (kPa). The M probe was used for all measurements. As an indicator of variability, the ratio of the interquartile range of the LSM to the median LSM was measured. Only the cases with at least 10 valid TE acquisitions, a success rate of ≥60%, and an interquartile range to the median LSM ratio of <0.3 were considered reliable and included in the statistical analysis. The cutoff values of the LSM for the diagnosis of liver fibrosis in this study were 7.2 kPa for F ≥ 2, 9.4 kPa for F ≥ 3, and 12.2 kPa for F = 4. These cutoff values were determined according to previously published data from a meta-analysis (14).

Regression of liver fibrosis was defined as F0–F1 (LSM < 7.2 kPa) at the last follow-up visit for patients with F2–F3 (LSM ≥ 7.2 kPa and LSM < 12.2 kPa) at baseline, or F0–F1–F2 (LSM < 9.4 kPa) for patients with F4 (LSM ≥ 12.2 kPa) at baseline.

### Gender and reproductive variables

Information on menarche and menopause was obtained from the women who participated in the study using face-to-face clinical interviews and questionnaires. Self-reported data regarding predefined reproductive factors have been shown to be highly reproducible in epidemiological studies (16, 17). Questions regarding the menopausal status included asking whether the menstrual periods had ceased permanently and, if they had, at what age and for what reason (e.g., natural menopause or surgical menopause after hysterectomy and bilateral oophorectomy). Menopausal status was defined as no menstrual period for the previous 12 months (18). Age at menarche was defined as the age of the first menstrual period.

The duration of reproductive life, or reproductive age, was determined by subtracting the age at menarche from the age at menopause. The length of menopause, or time from menopause, was calculated as the age at the time of enrollment to the study minus the self-reported age at menopause.

### Other clinical and demographic variables

Further demographic information obtained from the study participants included age, gender, body weight, height, body mass index (BMI) (calculated as weight in kilograms divided by the height in meters squared), waist circumference, and hip circumference. Obesity was defined as BMI ≥ 28 kg/m^2^ according to the Chinese criteria (19). Laboratory data, including serum ALT, HBeAg status, and level of HBV DNA, were all obtained from our central laboratory information system.

Smoking was categorized as with or without a smoking history (20). Alcohol use was categorized as with a significant drinking history (>20 g/d) or without. Patients were identified as having type 2 diabetes from the diagnosis recorded in the electronic medical records or a fasting blood glucose level of >126 mg/dL on at least 2 occasions before enrollment to the study. The diagnosis of hypertension was also based on the electronic medical records. The controlled attenuation parameter was obtained simultaneously through TE examination for the evaluation of hepatic steatosis. The cutoff value of the controlled attenuation parameter was 248 dB/m, which was used to define hepatic steatosis (21).

### Statistical analysis

Data for continuous variables were presented as the median with minimum to maximum range, whereas data for categorical variables were presented as the percentage. Female and male cases were matched by age in a 1:1 ratio in a matched case-control study. The Student *t* test or nonparametric tests were used to compare continuous variables. The χ^2^ test was used to compare categorical variables. Changes in LSM during antiviral therapy were assessed with a paired-samples *t* test. Different modeling approaches were used to assess the associations between the categories of gender, menopause, and age at menarche, and the risk of advanced fibrosis. These approaches considered potential confounders. Odds ratios (ORs) were estimated from the models and presented with 95% confidence intervals (CIs). All statistical tests were 2-sided. A *P* value of <0.05 denoted statistical significance. Statistical analysis was performed using SPSS 22.0 software (SPSS Inc, Chicago, IL).

## RESULTS

### Patient characteristics according to gender and menopausal status

There were 716 women who met the study inclusion criteria. The menopausal status was determined in 121 patients (16.9%). After case-control matching, 716 male patients were also included in the prevalence analysis (Figure 1). The clinical and biochemical characteristics of the enrolled participants were grouped as premenopausal women (PRW) (n = 595), postmenopausal women (POW) (n = 121), men who were age-matched with the PRW (n = 595), and men who were age-matched with the POW (MPOW) (n = 121), as shown in Table 1.

**Table 1.**
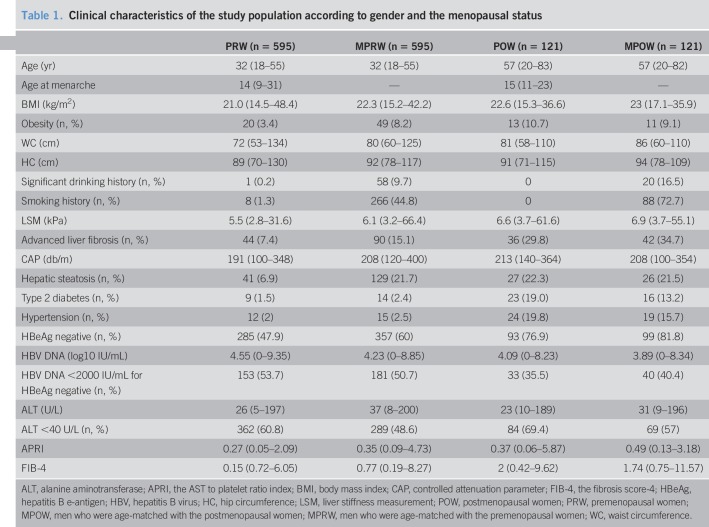
Clinical characteristics of the study population according to gender and the menopausal status

### The association between menopause and advanced liver fibrosis

To assess the relationship between advanced liver fibrosis and reproductive factors, the PRW and POW included in the study were divided into 2 groups, namely women without advanced fibrosis (F0–F2) and women with advanced fibrosis (F3–F4). Table 2 summarizes the demographic and reproductive characteristics of these 2 groups. For POW, the 2 groups had similar age at menopause (49 vs 50 years, *P* = 0.544), reproductive age (35 vs 34 years, *P* = 0.135), and length of menopause (5 vs 10 years, *P* = 0.211).

**Table 2.**
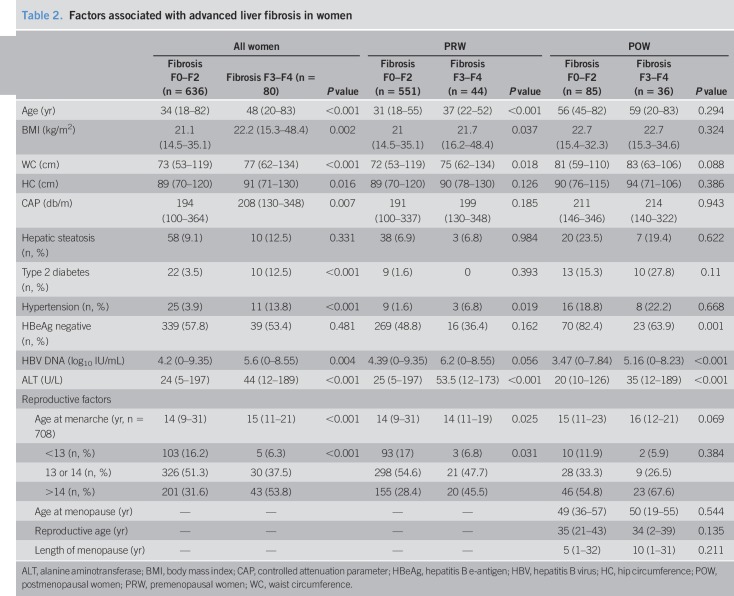
Factors associated with advanced liver fibrosis in women

Multiple regression models were constructed to assess whether the menopausal status was independently associated with advanced fibrosis (Table 3). The results showed that the menopausal status was associated with an increased risk of advanced fibrosis (unadjusted OR = 5.304; 95% CI: 3.299–8.711; *P* < 0.001). In the initial multivariate model, after adjusting for age, the association between the menopausal status and advanced fibrosis was mildly reduced; however, it remained statistically significant (adjusted OR = 3.653; 95% CI: 1.57–8.501; *P* = 0.003). In the second multivariate model, after additional adjustment for metabolic factors (e.g., BMI, diabetes mellitus, and hypertension), the risk was also significantly increased (adjusted OR = 3.722; 95% CI: 1.589–8.954; *P* = 0.003). Further adjustment for markers of liver injury (e.g., ALT) in the third model (adjusted OR = 5.8; 95% CI: 2.302–14.615; *P* < 0.001), and viral factors (e.g., HBeAg status and HBV DNA) in the fourth model (adjusted OR = 8.833; 95% CI: 3.026–25.783; *P* < 0.001), showed that the association between the menopausal status and advanced fibrosis was statistically significant.

**Table 3.**
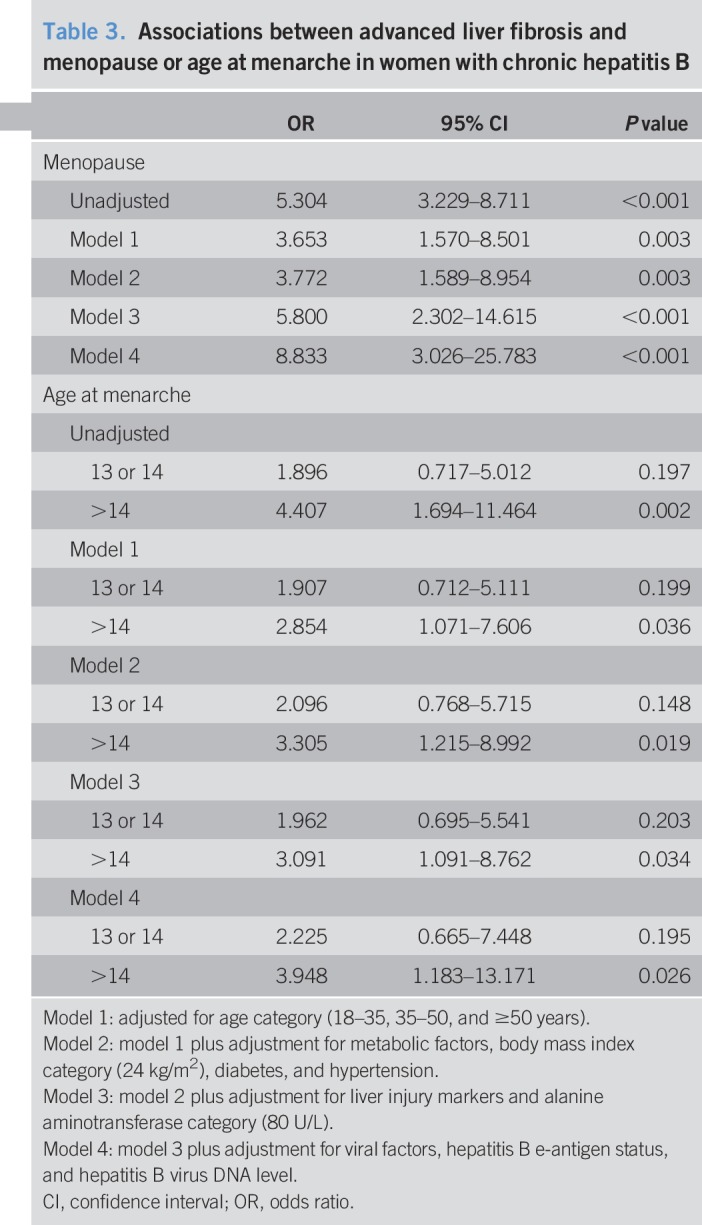
Associations between advanced liver fibrosis and menopause or age at menarche in women with chronic hepatitis B

### The association between age at menarche and advanced liver fibrosis

Compared with women without advanced fibrosis, those with advanced fibrosis had an older age at menarche (15 vs 14 years, respectively; *P* < 0.001) (Table 2). The prevalence of menarche at an age of >14 years in patients without and with advanced fibrosis was 31.6% and 53.8%, respectively (*P* < 0.001).

**Figure 2.**
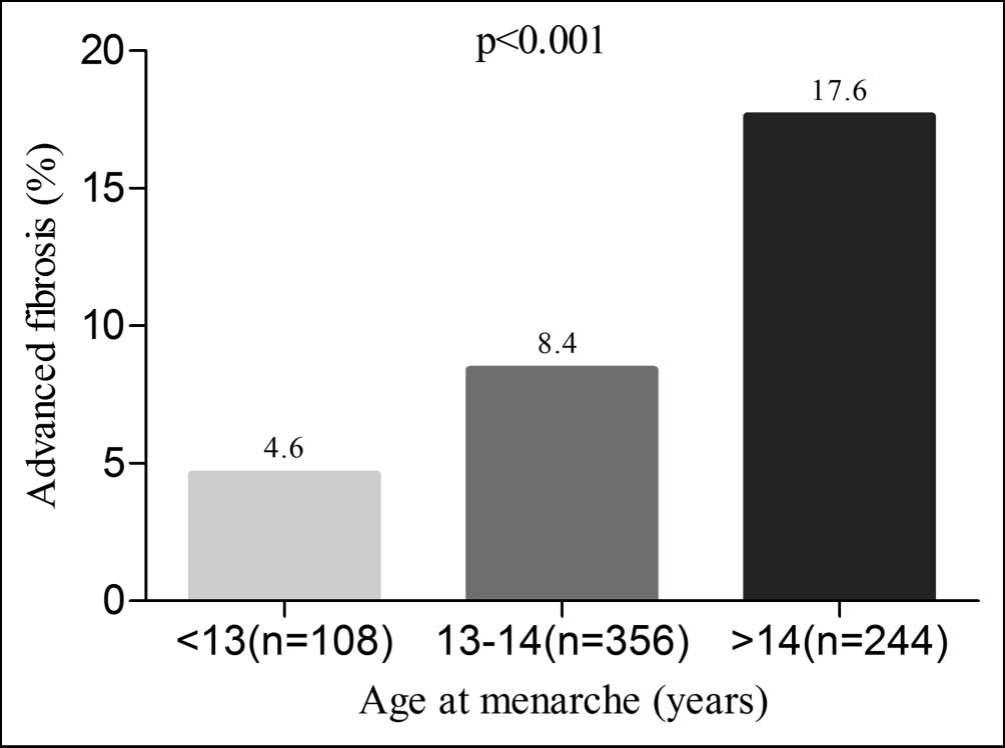
Prevalence of advanced liver fibrosis stratified by age at menarche.

The association between advanced fibrosis and age at menarche is shown in Figure 2. Patients were categorized into 3 groups based on their age at menarche, namely <13, 13 or 14, and >14 years. The prevalence of advanced fibrosis increased in parallel with the age at menarche (4.6%, 8.4%, and 17.6%, respectively; *P* < 0.001).

The unadjusted OR for advanced fibrosis was 4.407 (95% CI, 1.694–11.464) in the late menarche (>14 years) group (*P* = 0.002), using the early menarche (<13 years) group as the reference group (Table 3). Late menarche (>14 years) continued to be significantly associated with advanced fibrosis even after adjustment for age (adjusted OR = 2.854; 95% CI: 1.071–7.606; *P* = 0.036) and remained significant after additional adjustments for metabolic factors (adjusted OR = 3.305; 95% CI: 1.215–8.992; *P* = 0.019), liver injury markers (adjusted OR = 3.091; 95% CI: 1.091–8.762; *P* = 0.034), and viral factors (adjusted OR = 3.948; 95% CI: 1.183–13.171; *P* = 0.026).

### The association between gender and advanced liver fibrosis

Previous studies have shown that viral factors (HBeAg status and HBV DNA), ALT levels, the presence of obesity, smoking, and alcohol consumption were associated with the severity of liver fibrosis in patients with CHB (9, 22, 23). Importantly, in the present study, these factors also showed significant differences between men and women (Table 1). Therefore, we examined the relationship between gender and advanced fibrosis using multivariate regression, adjusting for a comprehensive list of confounding factors. Furthermore, the effect of female gender was assessed according to menopausal status (Table 4). In the men who were age-matched with the PRW group, the unadjusted OR for advanced fibrosis was 2.232 (95% CI: 1.526–3.264), using the PRW group as the reference group. This association remained significant in 4 different multivariate regression models, although the association was minimally diminished (OR = 1.721–2.021, all *P* < 0.05). However, in the MPOW group, the unadjusted OR for advanced fibrosis was 1.255 (95% CI: 0.731–2.155), using the POW group as the reference group. This association remained nonsignificant in 4 different multivariate regression models (all, *P* > 0.05).

**Table 4.**
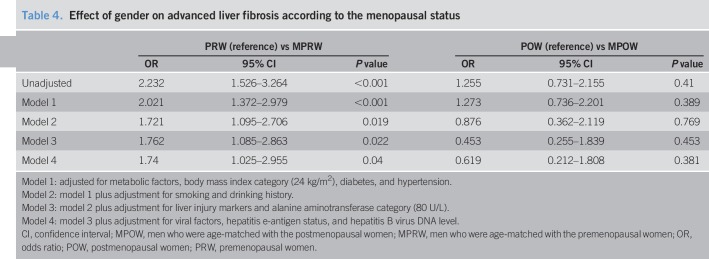
Effect of gender on advanced liver fibrosis according to the menopausal status

### The association between menopause and the regression of liver fibrosis

A total of 544 patients (76%) from the prevalence cohort were excluded from the study because of the absence of liver fibrosis at baseline. The remaining 50 patients who began regular antiviral therapy with more than 1 TE measurement were enrolled in the analysis of the regression of liver fibrosis (Figure 1).

**Figure 3.**
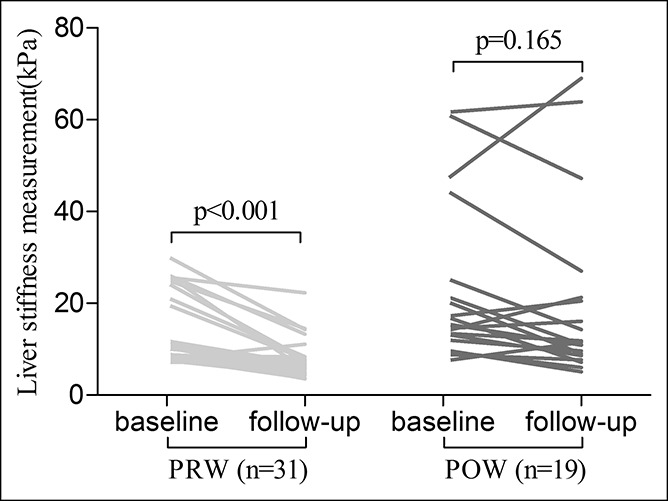
Liver stiffness measurements before and during antiviral therapy according to the menopausal status. POW, postmenopausal women; PRW, premenopausal women.

The POW group (n = 19) (baseline mean: 22.9 kPa vs follow-up: 19.9 kPa; *P* = 0.165) had a slower decline in the LSM vs the PRW group (n = 31) (baseline mean: 13.3 kPa vs follow-up: 7.5 kPa; *P* < 0.001) (Figure 3). The treatment response of 50 female patients at the end of follow-up was shown in Table 5. The POW group had fewer women with the regression of liver fibrosis during antiviral therapy compared with the PRW group (26.3% vs 74.2%, respectively; *P* < 0.001). In the PRW group, prescribed antiviral therapy included entecavir (n = 20), peginterferon alfa-2a (n = 7), tenofovir (n = 3), and telbivudine (n = 1). In the POW group, prescribed antiviral therapy included only entecavir. The median follow-up and treatment time in the PRW and POW groups was similar (*P* > 0.05). Comparison of the POW and MPOW groups revealed a similar prevalence of undetectable HBV DNA and normal ALT (<40 U/L) at the last follow-up.

**Table 5.**
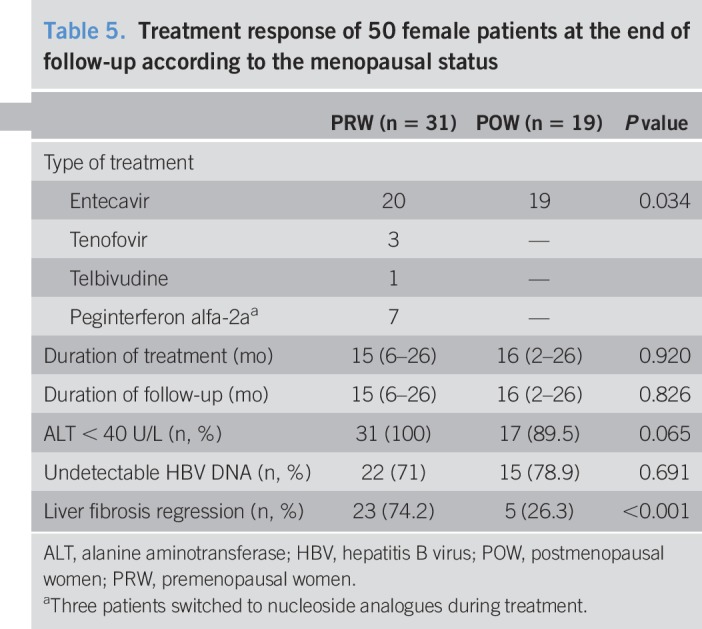
Treatment response of 50 female patients at the end of follow-up according to the menopausal status

The association between age at menarche and the regression of liver fibrosis was not statistically significant (data not shown).

## DISCUSSION

This large, prospective, cross-sectional study demonstrated several novel findings. First, we found that the menopausal status and late menarche were independently associated with an increased risk of advanced liver fibrosis in women with CHB. Second, the protective effect of female gender against developing advanced fibrosis was observed only in PRW, which disappeared in POW. Finally, the menopausal status delayed the regression of liver fibrosis after the initiation of antiviral therapy.

The finding that menopausal status was associated with an increased risk of advanced fibrosis is consistent with that reported by several other studies investigating liver diseases, including NAFLD and HCV infection (1, 2, 4). We acknowledge that age, which is closely correlated with the postmenopausal status, is associated with the progression of liver disease in CHB (24). However, the association between the postmenopausal state and advanced fibrosis remained statistically significant, even in age-adjusted and fully adjusted models. On the other hand, when the effect of gender on liver fibrosis in PRW and POW was independently assessed, the protective effect of female gender against the development of advanced fibrosis was observed only in PRW. These results suggest that the progression of fibrosis was rapidly accelerated after menopause and was likely to be due to the postmenopausal status, not just aging.

Previous studies have shown that ALT levels, the presence of obesity, smoking, and alcohol consumption were associated with the severity of liver fibrosis in patients with CHB (9, 22, 23). The association between gender and liver fibrosis for PRW and age-matched men was minimally diminished after adjusting for the above-mentioned factors in multiple regression models. Therefore, gender alone cannot explain the differences in fibrosis between PRW and age-matched men in this study population.

In the present study, we did not observe that age at menopause, reproductive age, and length of menopause were associated with advanced fibrosis. However, in contrast to our results, a previous study showed that age at menopause (<40 years) and duration of menopause were significant risk determinants for the severity of liver fibrosis in NAFLD (5). Several factors may explain these discrepancies, including differences in the study populations and the etiology of chronic liver disease between CHB and NAFLD. Notably, in the present study, only 3 women underwent menopause at an age of <40 years.

The relationship between age at menarche and the development of liver disease is not well characterized. The relationship between age at menarche and CHB was previously reported in a long-term cohort study, showing that an earlier onset of menarche was associated with significantly higher incidence of spontaneous HBeAg seroconversion, decreased HBsAg titers, and an increased rate of HBV clearance (13). Also, several epidemiological studies have reported that early menarche is associated with an increased risk of NAFLD (25, 26). Our findings suggest that it may be important to consider age at menarche when establishing surveillance strategies among women with CHB, especially from puberty. The association between age at menarche and liver fibrosis in patients with other causes of chronic liver disease requires further study.

Several previously published studies have investigated the reduction in liver fibrosis during antiviral treatment in patients with CHB using TE (27–29). However, these previous studies have not reported the effect of menopause on liver fibrosis. The data from the present study showed that the menopausal status is associated with reduced likelihood of the regression of fibrosis in patients with CHB. However, further studies are warranted to investigate whether estrogen replacement therapy may lead to higher rates of the regression of liver fibrosis during antiviral therapy.

The mechanisms underlying the effect of menopause on HBV-associated liver fibrosis remain unknown. Given the protective effects of estrogens in hepatic injury (30, 31), fibrogenesis (32, 33), and HBV infection (12, 34), we hypothesize that a state of estrogen deficiency may be associated with the progression of liver fibrosis. There are several lines of evidence to support this hypothesis. First, previous experimental studies have shown the protective effect of estrogen on liver injury, regardless of the cause of injury (30, 31). Second, estrogen has been shown to reduce collagen synthesis in male rats exposed to dimethylnitrosamine (32). Also, estradiol has been shown to inactivate the downstream transcription processes involved in the expression of transforming growth factor-β1 and activation of hepatic stellate cells, which are key factors in hepatic fibrogenesis (33). Finally, estrogen can inhibit the transcription of HBV genes by upregulating estrogen receptor-α, which interacts with and alters the binding of hepatocyte nuclear factor-4α to the HBV enhancer I (12). Moreover, estrogen may be involved in the immune response to HBV infection. The prevalence of CD107+ natural killer cells in the female liver is significantly greater than that reported in men and is correlated with the plasma levels of estradiol in CHB (34). Future studies are required to characterize the mechanistic pathways involved in the association between the menopausal status and liver fibrosis in CHB.

This was the first study to assess the influence of reproductive factors on HBV-associated liver fibrosis. The study included consecutively recruited women who were age-matched with men and studied during the same period. This eliminated possible bias associated with patient selection. Furthermore, we applied different multivariate models to evaluate the effects of menopause, age at menarche, and gender on advanced fibrosis, which helped to validate the findings of this study.

This study was also characterized by limitations. First, data regarding the effects of reproductive factors on advanced fibrosis were derived from a cross-sectional study, which limited our ability to determine the causality of temporal associations. Second, the absence of histologic confirmation using liver biopsy as a reference standard limits the validity of the present results. Third, our cohort did not contain sufficient numbers of POW who were treated with estrogen replacement therapy, and there was no analysis of the possible beneficial impact of estrogen replacement therapy on liver fibrosis in POW. Finally, the XL probe designed for use in obese patients is not available in our study. The invalid measurements performed by the L probe in several morbidly obese patients cannot further performed by the XL probe.

In conclusion, the menopausal status and late menarche increased the risk of advanced liver fibrosis in women with CHB. In addition, the menopausal status delayed the regression of fibrosis during antiviral therapy. The protective effect of female gender against the development of advanced liver fibrosis was no longer present in POW. Future studies are warranted to study the mechanism of reproductive factors in CHB.

## CONFLICTS OF INTEREST

**Guarantor of the article:** Jinjun Chen, MD, PhD.

**Specific author contributions:** M.X. contributed to the design of the study, patient recruitment, data acquisition, data analysis and interpretation, statistical analysis, and writing of the manuscript; J.L. contributed to patient recruitment, data acquisition, data analysis and interpretation, and statistical analysis; S.Y., F.Z., Y.J., J.L., and Q.W. contributed to patient recruitment and acquisition of data; Q.H., R.J., F.Z., and W.W. contributed to patient recruitment; J.C. contributed to the study design, patient recruitment, data analysis and interpretation, critical revision of the manuscript, funding provision, and writing of the manuscript; and J.H. supervised the study and submission for publication. All authors approved the final version of the manuscript.

**Financial support:** This study was supported in part by grants from the National Science and Technology Major Project (2018ZX10723203), the National Natural Science Foundation of China (81270533 and 81470038), the National Key Research and Development Program of China (2017YFC0908100), the Key Scientific and Technological Program of Guangzhou City (201508020262), the Department of Science and Technology of Guangdong Province (2014B020228003 and 2015B020226004), the Clinical Research Startup Program of Southern Medical University (LC2016PY005), and the National High Technology Research and Development Program (2012AA022605).

**Potential competing interests:** None to report.

**Study Highlights**

**WHAT IS KNOWN**

✓ The severity of liver fibrosis associated with chronic hepatitis C and NAFLD is increased in POW.
✓ In patients with CHB, the female gender is a protective factor against disease progression.

**WHAT IS NEW HERE**

✓ The onset of menopause and late menarche independently increased the risk of advanced liver fibrosis in women with CHB.
✓ In women with CHB, the protective effect of the female gender against developing advanced liver fibrosis disappeared after menopause.
✓ In women with CHB, the postmenopausal status delayed the regression of liver fibrosis after the initiation of antiviral therapy.

**TRANSLATIONAL IMPACT**

✓ It is important to consider menopause status and age at menarche in establishing surveillance strategies among CHB females. Estrogen may have the potential preventive and/or therapeutic effects in CHB.
